# A Hybrid Convolutional Neural Network Model for Diagnosis of COVID-19 Using Chest X-ray Images

**DOI:** 10.3390/ijerph182212191

**Published:** 2021-11-20

**Authors:** Prabhjot Kaur, Shilpi Harnal, Rajeev Tiwari, Fahd S. Alharithi, Ahmed H. Almulihi, Irene Delgado Noya, Nitin Goyal

**Affiliations:** 1 Chitkara University Institute of Engineering and Technology, Chitkara University, Rajpura 140401, Punjab, India; prabhjot.k@chitkara.edu.in (P.K.); shilpi13n@gmail.com (S.H.); 2 Department of Systemics, School of Computer Science, University of Petroleum and Energy Studies, Dehradun 248007, Uttarakhand, India; errajeev.tiwari@gmail.com; 3 Department of Computer Science, College of Computers and Information Technology, Taif University, Taif 21944, Saudi Arabia; f.alshalawi@tu.edu.sa (F.S.A.); a.almulihi@tu.edu.sa (A.H.A.); 4 Higher Polytechnic School/Industrial Organization Engineering, Universidad Europea del Atlántico, 39011 Santander, Spain; irene.delgado@uneatlantico.es; 5 Department of Project Management, Universidad Internacional Iberoamericana, Campeche 24560, Mexico

**Keywords:** convolutional neural network, COVID-19, disease detection, InceptionV4, SVM, chest XR images

## Abstract

COVID-19 declared as a pandemic that has a faster rate of infection and has impacted the lives and the country’s economy due to forced lockdowns. Its detection using RT-PCR is required long time and due to which its infection has grown exponentially. This creates havoc for the shortage of testing kits in many countries. This work has proposed a new image processing-based technique for the health care systems named “C19D-Net”, to detect “COVID-19” infection from “Chest X-Ray” (XR) images, which can help radiologists to improve their accuracy of detection COVID-19. The proposed system extracts deep learning (DL) features by applying the InceptionV4 architecture and Multiclass SVM classifier to classify and detect COVID-19 infection into four different classes. The dataset of 1900 Chest XR images has been collected from two publicly accessible databases. Images are pre-processed with proper scaling and regular feeding to the proposed model for accuracy attainments. Extensive tests are conducted with the proposed model (“C19D-Net”) and it has succeeded to achieve the highest COVID-19 detection accuracy as 96.24% for 4-classes, 95.51% for three-classes, and 98.1% for two-classes. The proposed method has outperformed well in expressions of “precision”, “accuracy”, “F1-score” and “recall” in comparison with most of the recent previously published methods. As a result, for the present situation of COVID-19, the proposed “C19D-Net” can be employed in places where test kits are in short supply, to help the radiologists to improve their accuracy of detection of COVID-19 patients through XR-Images.

## 1. Introduction

In 2019, a new virus known as COVID-19 emerged, which was caused by a coronavirus strain known as “SARS-CoV-2”. When the WHO labeled the COVID-19 outbreak a pandemic in March 2020, the situation became a worldwide health crisis. COVID-19 is a worldwide health emergency that resulted in over 45 million recorded illnesses and a death rate of 1.2 million, and the majority of the world is still in danger [1]. Prognostic forecasts are a prerequisite to enhance and coordinate the care of a patient during the COVID-19 pandemic. The COVID-19 makes antibiotics ineffective in treating patients, complicating the infection if their immune system is poor [2]. Recently, several pharmaceutical corporations and research institutions have developed vaccines, and many countries commence vaccination; however, the hope that this pandemic could be finally defeated has been jeopardized by the appearance of several mutants of higher transmissible. This backs the pandemic’s fighting to square one; indeed, some nations face the second or third waves of COVID-19 while others reinforced curfew and lockdown as precautionary solutions. The lack of holistic treatment fuels the urgent need for fast, low-cost, and reliable tools to diagnose the disease as early as possible. The large and complex symptoms displayed on patients, however, pose a significant challenge for rapid diagnosis of COVID-19 [3,4]. Although the majority of patients have mild symptoms (fever, cold, cough); some may have more serious signs (breathing problem, muscle weakness, anxiety, depression, and joint pain) or even no symptoms at all as asymptomatic patients.

To isolate and classify all identified viral genetic variants of the disease, RT-PCR involves taking samples from the nose or throat and testing them chemically. The RT-PCR test has a high false-negative ratio, with long duration to generate results and the limited convenience of its kits in some states. Several study groups report [1] that, the rate is up to as high as 61%; other sources indicate that RT-PCR has a maximum accuracy of 0.8 [5] and can only be obtained on or after the eighth day of symptom onset. Chest radiographs are used as an adjunct or alternative diagnostic method to address the crucial limitations of the RT-PCR test. Rather than determining the infectious status at a single time point, as RT-PCR does, the useful information encoded in these images helps physicians to monitor disease progression [6]. This qualifies them for illness diagnosis and assessment. The peripheral zone of the lungs has several small patchy shadows and interstitial changes; however, as the disease progresses, multiple ground-glass opacities (GGO) and infiltration occur in both lungs, and pulmonary convergence is regarded as a sign of a serious case. According to estimates for the time and precision of the manual test, a cardiothoracic fellowship-trained radiologist examines an X-ray image in about a minute on average, with an average diagnostic accuracy of about 78% [7,8]. This is a significant amount of diagnosis time, particularly when you realize that about 35,000 confirmed cases are recorded every day around the world. This can offer more time for the treatment of suffering patients with such an infectious disease which is declared as a pandemic by WHO.

The “polymerase chain reaction (PCR)” or “computed tomography (CT)” images have been used for the diagnoses. Decision trees have been used for intelligent decision-making in various fields since the analysis of image processing. Similarly, improved software tools can assist physicians in understanding and studying images to obtain another option, which can serve as validation of the radiologist’s diagnosis and treatment protocols, thus increasing the patient’s survival rate [9]. The lack of a comprehensive dataset that belongs to various forms of infection intensity poses a significant challenge for this problem statement. The effectiveness and benefits of AI in medicine have been shown several times. The transfer learning approach or pattern recognition technique has been used for the recognition of COVID-19 disease and creating a better system using X-Ray imagery [10]. The accuracy of 92.49% is achieved using AI techniques for the detection of COVID-19 with chest X-ray images [11,12]. As the virus spreads through contact of one person with another, it has wreaked havoc on human lives, putting a strain on the public healthcare system and global economies due to lockdowns at places. Preventing viral propagation necessitates detection of positive cases at an early stage and prompts treatment of disease-ridden individuals. Every year, as technology advances in the AI industry, the contribution of automated systems to provide better health care predictions has been improved. For time-saving and the reduction of manpower, recently, artificial intelligence models have been developed for the automatic diagnosis of illness.

Various researchers have proved that the COVID-19 recognition method is based on VGG-16 [13] model transfer learning. With 96% accuracy, this method helps in distinguishing between healthy and illness X-ray pictures. The use of COVIDX-Net [14] model employs different neural network models for binary classification [15] both CNN models have provided the same accuracy of 90%. The COVID, normal, and pneumonia CRIs images can be classified using a transfer learning technique in binary and three-class categorization. When MobileNet v2 [16] was employed, classification accuracies for binary and 3-class classification were 97.4% and 92.85%, respectively. The detection of COVID 19 patients from CRIs images has also been employed using the deep learning (DL) ResNet-152 architecture [17]. DL employs the synthetic minority oversampling technique (SMOT) for balancing some unequal vertices. The model achieved an accuracy of 97.7% with XGB and 97.3% with the RF classifier.

Using “computed tomography” CT images of COVID infected persons, several methods have proposed the multi-view, single-view fusion models for the identification and classification of disease [18]. The validation and training of such multi-view model are done on some regions such as coronal, axial, and sagittal. The accuracy achieved using artificial neural network (ANN) and deep neural network (DNN) models were 94.10% and 95.84% respectively with a proposed hybrid framework of feature selection and feature generator technique for multi-view classification and detection of disease [4]. Another model with a union of DCNN and capsule neural network (CapsNet) [19] for corona pneumonia detection can be employed for better detection results with an achieved accuracy of 90%, “F1-score” of 90.9%, and the “sensitivity” of 96%. Another option is to apply a two-stage transfer learning model [20] for the detection of COVID-19 CT scanned images. These pre-trained models can achieve improved efficiency by applying ensemble methods. Such a proposed model gives assistance accuracy of 90.82% with an “F1 score” of 85.86%. Using DL models some researchers have achieved good accuracy while some others lack due to interpretation. Among all classification models, the ResNet50 gives an average accuracy of 97% [21]. The pre-processed images followed by state-of-art DL models for the classification of data have been suggested by some researchers and the accuracy of positive and negative patients has also been calculated [22].

When the dataset is unbalanced, the training strategies can aid the network in learning. According to some authors, Inception V4 [23] network can achieve an average accuracy of 99.50 percent for detecting COVID-19 instances, with an overall accuracy of 91.4 percent for all groups. The “COVIDX-Net” [24] combines multiple pre-trained CNN networks with a binary classifier to provide quick diagnoses for COVID19 from X-ray images. VGG19, DenseNet201, InceptionV3, ResNetV2, InceptionResNetV2, Xception, and MobileNetV2 are the seven models that make up COVIDX-Net. Among them, each model has been trained and tested separately. DeTraC [25] uses CNN-based pre-trained models, “ImageNet” and “ResNet”, to extract local features in each image separately and then transfer them to the decompose layer. Table 1 summarizes some of the key findings reported by the peer works.

Some of the authors suggested different deep learning models such as ResNet-101, ResNet-50, DenseNet-169, DenseNet-201 [26], ResNet-18 [29], AlexNet [31], CNN [32], for the diagnosis of disease using CT images, CXR images but very few have suggested bi-level programming Stackelberg [33], optimizer algorithm [34] for multi-classes. For healthcare operations some authors suggested different heuristics and metaheuristics models [35], multi-depot routing model [36], logistic problems [37], and some suggested mesenchymal stem cell (MSC) therapy [38] treatment. It is observed from the survey that the maximum researchers’ have used very few images for the detection of COVID-19 infection using chest X-Ray images, except the one researcher who has trained and tested 5856 images [31]. In addition, for the classification of COVID-19, researchers have worked on a maximum of two or three classes of the image dataset. Thus, the requirement of a hybrid model arose with four classes of image dataset for better accuracy and detection.

The model proposed in this work, i.e., C19D-Net is introduced for the diagnosis of Chest XR images disease which is better than other deep learning models in terms of accuracy and time. Manual disease detection and categorization take a long time to complete, and pathologists can face observer variability issues when grading the types of diseases. Many researchers have recently presented various strategies for classifying COVID-19 from XR images. The majority of studies have used Image Processing methods to identify COVID-19 from patient chest XR. Some detect the COVID-19 using binary classes; others use three different classes, and a few uses four classes. Our aim is to use all the different classes or combinations of classes for the detection of disease. The classifier used in this paper gives better accuracy in less time as compared to other classifiers which take more time. As observed from various papers more instances surface every day, the demand for COVID-19 testing kits has increased, and many poor countries are experiencing a shortage of testing kits, also take more time to detect the result of COVID-19. In this case, the current research uses deep learning techniques that are useful in detecting COVID-19, and give crucial facts regarding the COVID-19 virus-caused sickness.

## 2. Materials and Methods

### 2.1. Dataset for Experiment

To evaluate the performance of C19D-Net, the chest XR images (dataset) are collected from two different repositories. Firstly, the public Chest XR data sources collect from the GitHub database [39]. Then, for normal and pneumonia images data is collected from the Kaggle database [40]. These open repositories are updated regularly. The use of chest XR images to diagnose COVID-19 is reliable as it is more effective at excluding COVID-19 infection than it is separating it from other respiratory disorders. A physician’s radiological expertise may not always result in an accurate diagnosis. The nose, throat swabs, and blood tests, on the other hand, take a longer time to produce results.

**Figure 1:**
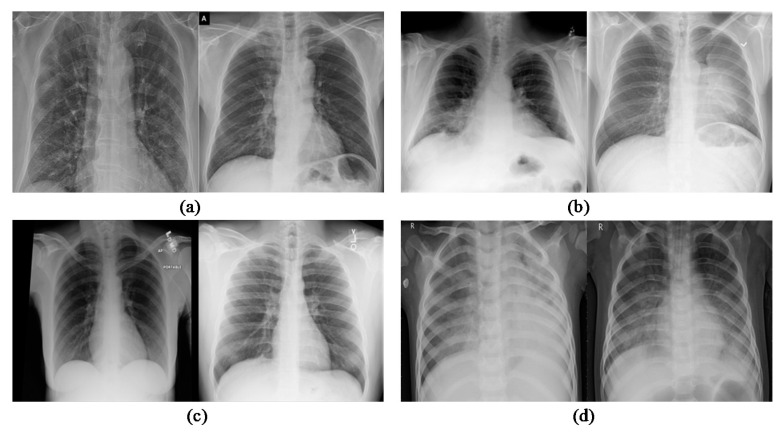
Pictorial representation of (**a**) COVID-19 images, (**b**) bacterial_pneumonia images, (**c**) normal images, (**d**) viral_pneumonia images.

As a result, chest XR images play a vivacious part in the care and follow-up of hospitalized patients. The main inclusion of the dataset in this paper is the XR images of the chest which are pre-processed. The XR images of more than 15-year-old patients are considered rest others are excluded. Table 2 and Table 3 summarize the prepared dataset for four-class, three-class, and Table 4 précises the dataset related to two-class. All the images are pre-processed to the same size. In two-class, three-class, and four-class groups we categorize 400 images are for COVID-19 and 600 for normal images according to the tables. Bacterial pneumonia and viral pneumonia images for four-class are 450 and 450 in number respectively. However, for three-class the combined pneumonia images are 900. The dataset representation is shown in Figure 1.

### 2.2. Methodological Contribution

In this study, a proposed C19D-Net model is designed for the detection of COVID-19 disease from all 4 classes (“COVID-19”, “viral pneumonia”, “normal”, and “bacterial pneumonia”) of the dataset, both separately and in combination, with the possibility of detecting the stage of the disease. Firstly, the input photos which are of different sizes must be pre-processed to equal size of 224 × 224 × 3 pixels for improving accuracy as a normalization step for the proposed DL model. The performance with C19D-Net implies better with InceptionV4—as it has 43M parameters, 230 layers, and scaling factor varies from 0.1 to 0.3 with an error rate of 0.001. Extracted features are then fed into SVM classifier for the better classification of disease. Performing an empirical investigation of the competitive deep learning image classifiers for identifying COVID-19 disease using conventional chest XR images that are less expensive than other imaging modalities.

### 2.3. Proposed C19D-Net Model

Deep neural networks are estimable in image processing tasks. This has resulted in a variety of applications for industries, including manufacturing, agriculture, and medical disease diagnostics. The valuable and robust properties retrieved from input photos give these convolutional neural network (CNN) networks their notoriety. Hence, the chest XR medical images are also classified with the deep learning models into bacterial pneumonia, normal, viral pneumonia, and COVID-19. Some pre-trained CNN models VGG16, VGG19, Xception, DenseNet, and ResNet have limited parameters and fewer hidden layers which are used for classifying the data. In addition, they are not more efficient in terms of efficiency, accuracy, error rate, model size. The proposed model contains the feature extractor InceptionV4 model and classifier SVM. With 41 million parameters, 229 Convo layers, and 1 fully connected layer. For difficult computational tasks, DL architectures have been applied, and the output has been produced with lesser time complexity. CNN was one of the earliest neural networks to be developed [30]. CNN refers to a collection of networks that may be used to divide an image into several categories. The multilayer perceptron is the foundation of CNN. The CNN algorithm has been used to successfully categorize images. Images are turned into multidimensional matrices when they are fed into the CNN [31]. Further, the filters are used to extract a distinct pattern from the square region of pixels, and the number of steps in photos is filtered using activation maps.

**Figure 2:**
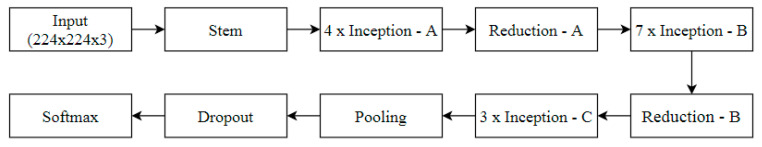
The Architecture of InceptionV4 is used for feature extraction.

Inception V4 is a CNN-based light design network. Some of the numerous building components that make up Inception are the dropouts, fully connected layer, and pooling layer with softmax serving as the loss function. Inception V4 has around 43M parameters. The input photos must be scaled to a size of 224 × 224 × 3 pixels. The training samples must be passed frequently for the training to enhance the accuracy of the classification that is referred to as steps of an epoch. Figure 2 shows the graphical representation of Inception V4. The input stems from 4 layers Inception-A to 7 layers Inception-B and then connects with 3 layers Inception-C and passes to pooling, dropout, and finally with softmax layer for extracting the features.

#### 2.3.1. Steps

The framework of C19D-Net includes three major steps in completing the clinical trial.

- **Step 1: Pre-processing**
  All Chest XR images have been collected in one dataset and scaled to a constant size of 224 × 224 × 3 pixels to be used in the proposed deep learning pipeline.
- **Step 2: Training and Validation**
  The training and validation process starts with dividing the images into an 80–20 ratio. This means the training phase contains 80% data and the testing phase contains 20% of data from the total. The Inception V4 produces features of the average pooling layer from the input image.
- **Step 3: Classification**
  All the features are extracted using Inception V4 from Chest XR images and then the multiclass SVM (MSVM) classifier is applied. The classification with the proposed C19D-Net model classifies the Chest XR images into multi-classes as “normal”, “viral pneumonia”, “COVID-19” and “bacterial pneumonia” with high precision as associated with other models or methods as discussed below in coming sections.

#### 2.3.2. Architecture

**Figure 3:**
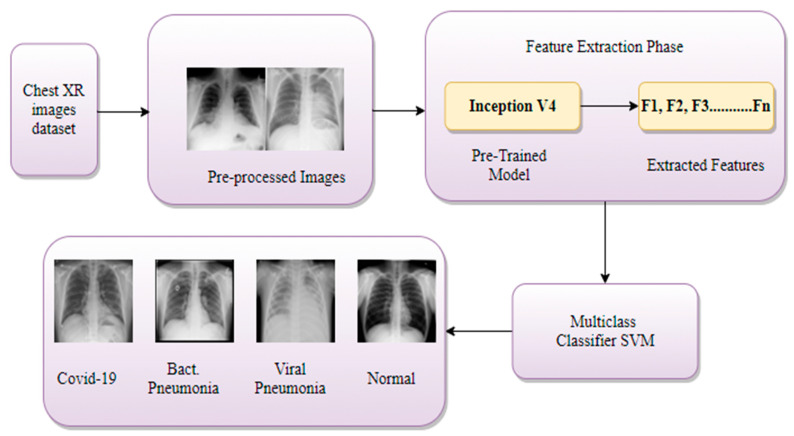
The architecture of the proposed C19D-Net.

The proposed C19D-Net model is based on the Inception V4 state-of-the-art model and uses chest XR images for feature extraction. Moreover, for the classification task, the multiclass SVM classifier was used. Figure 3 shows the architecture model of “C19D-Net”. A total of 1900 images of chest XR are collected. These images are divided into 4-class—COVID-19 images, viral pneumonia, bacterial pneumonia, and normal images. The C19D-Net model is trained on these images for the detection of accuracy from disease images. Firstly, the images are resized with pre-processing technique from 256 × 256 to 224 × 224 for getting better accuracy of the model. In the next phase, the features F1, F2, F3, …, Fn for all the images is extracted using a pre-defined InceptionV4 model. In the last stage, the featured images “COVID-19”, “bacterial pneumonia”, “viral pneumonia”, and “normal” are classified via support vector machine classifier which gives better accuracy as compared with other pre-defined classifiers as investigated through the literature.

#### 2.3.3. Training of Proposed Model (C19D-Net)

The COVID-19 has caused widespread concern around the world, yet only a small percentage of patient’s X-rays are available in the general public domain. For getting the absolute detection of disease a DL based architectures are used. Transfer learning techniques applied to CNN models, the suggested methodology includes classification and detection. The recognition of COVID-19 using chest XR images is done through three different scenarios. Each time the C19D-Net proposed model (Inception-V4 state-of-art CNN model) is trained on the Chest XR image dataset with the architecture shown in Figure 3. With the conversion of images into small channels, the model appropriately classifies the data. In a 4-class dataset, the C19D-Net model is trained to classify the data such as– “COVID-19”, “normal”, “viral pneumonia”, and “bacterial pneumonia”. In the 3-class dataset, the data is allocated into 3 classes/categories—pneumonia (viral pneumonia and bacterial pneumonia), COVID-19, and normal. In 2-class, the data is divided into two categories—COVID-19 and non-COVID. The normal and pneumonia images (both viral pneumonia and bacterial pneumonia) are pooled in the non-COVID class.

### 2.4. Statistical Analysis

This work has been used in the C19D-Net model a dataset of chest XR images, which has contributed to all statistical analysis parameters. The complete dataset has been statistically significant level *p*= 0.05 with size of 224 × 224. The different four classes of dataset specific exposure for the general population and impairment have been understood in the analysis of data. The comparison of the proposed C19D-Net model to the pre-trained Inception V4 model and achieved highest accuracy with different four class of dataset such as COVID-19 detection accuracy as 96.24%, 95.51% for 3-classes, and 98.1% for 2-classes. This work has been examining precision, recall, F1-Score, based upon confusion matrix of dataset variance. Therefore, to reduce the risk of work status and result, choose the confusion matrix.

## 3. Results

### 3.1. Metrics Evaluation

With the assessment of the performance of the suggested model and InceptionV4, the 4 most extensively used metric measures had been calculated: “*accuracy* (*ACC*)”, “pr*ecision*_value (PV)”, “re*call*_*value* (RV)”, and the “*F*1_*score* (FS)” as shown in Table 5, Table 6 and Table 7. Equation (1) shows the accuracy which is the ratio of the wide variety of real numbers to overall estimates. A wide variety of input samples; refers back to the potential of the version to discover the exact goal and locate the feasible goal of the model. The overall accuracy achieved with the C19D-Net model is shown in Table 8. The four-class dataset achieved an accuracy of 96.24% which is better as compared with pre-defined state-of-the-art models. The precision value in Equation (2) is the ratio of true positive values by the sum of all positive values. In the two-class dataset, the precision ranges from 96% to 98% as shown in Table 7.

As the number of images increases in one class the true positive value range increases. Equation (3) defines the Recall value which is the ratio of total positive with the total predicted value. It shows the percentage of the total predicted positive value. It ranges from 91% to 98%.

(1) $$Accuracy(ACC)\_C19D-\mathrm{Net}\;=\frac{TP+TN}{TP+TN+FP+FN}$$

(2) $$\mathrm{Pr}ecision\_C19D-\mathrm{Net}\;=\frac{TP}{TP+FP}$$

(3) Recall_C19D-Net =TPTP+FN

Pr*ecision* and re*call* have a harmonic mean which is calculated in *F*1_*Score* as represented in Equation (4). It considers both false positives and false negatives. As the number of images increases the v*alue* of the *F*1_*score* increases w.r.t pr*ecision value*. It ranges from 91% to 95% in three-class and four-class datasets respectively.

(4) F1_Score(FS)=2×Precision_Value×Recall_Value(RV)Precision_Value(PV)+Recall_Value(RV)

In the two-class comparison shown in Table 7, the F1-score and precision value increases as the range of specificity increases in the case of both C19D-Net and InceptionV4 along with the overall accuracy.

Table 8 shows the average precision, recall, specificity, F1-score, and accuracy of the proposed C19D-Net model with four classes, three classes, and two classes.

#### 3.1.1. 4-Class Evaluation Metrics Comparison

**Figure 4:**
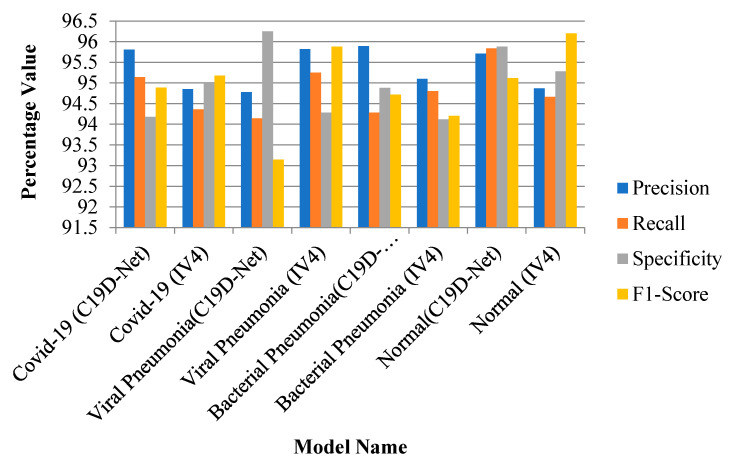
Evaluation performance of models with four-class.

In a four-class comparison, the accuracy achieved is 96.24% mentioned in Table 8. In evaluation metrics, the precision value increases with the increase of accuracy value. Figure 4 shows the metric measures that change with the effect of some images and with model accuracy.

#### 3.1.2. 3-Class and 2-Class Evaluation Metrics Comparison

**Figure 5:**
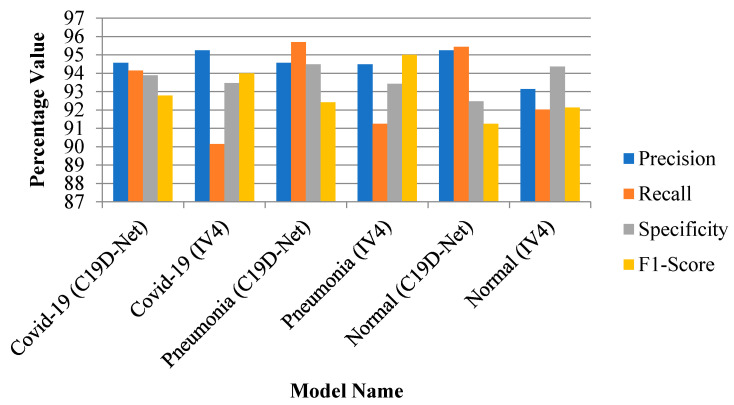
Evaluation performance of models with three-classes.

**Figure 6:**
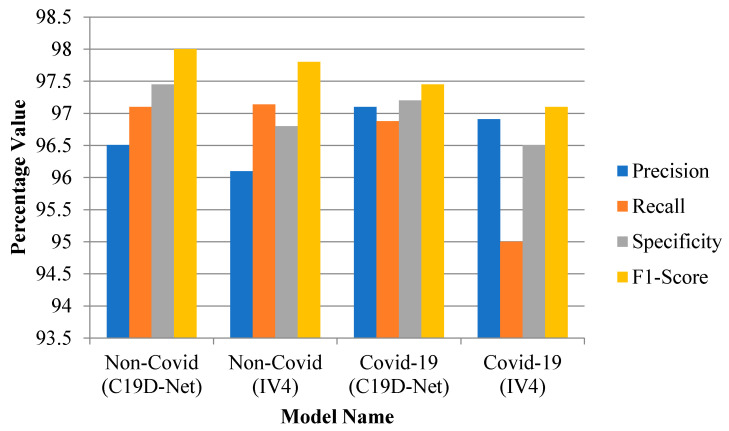
Evaluation performance of models with two-classes.

Figure 5 and Figure 6 shows the evaluation graph of three-class and two-class. The combination of pneumonia images shows the highest recall value with an increase of accuracy as compared with pre-defined models.

**Figure 7:**
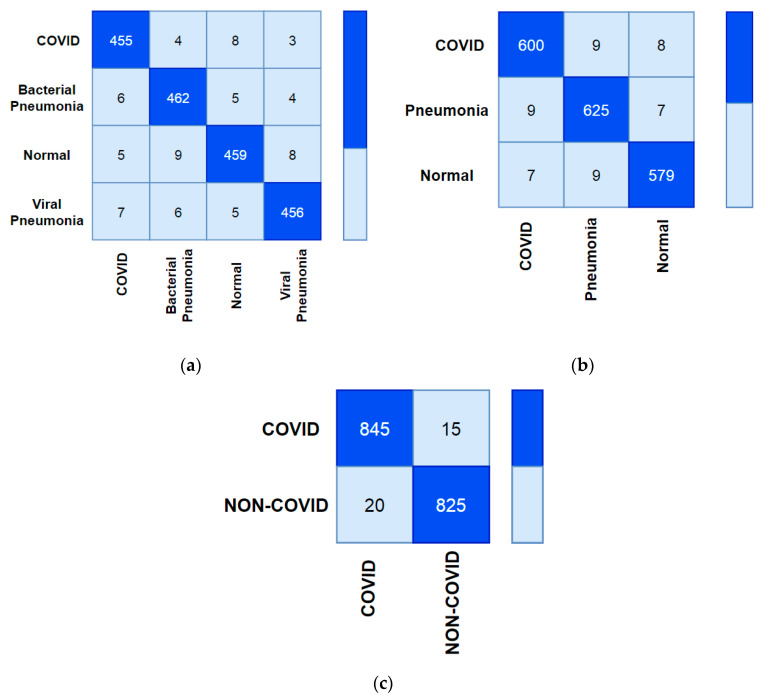
Confusion Matrix of C19D-Net model (**a**) four-class, (**b**) three-class, (**c**) two-class.

The overlapped matrix called the confusion matrix of all the classes is shown in Figure 7.

### 3.2. Experimental Result

The result of the four-class is shown in Table 5. The comparison of InceptionV4 with C19D-Net is shown in the table. The proposed C19D-Net model achieved the highest accuracy as compared with InceptionV4 shown in Table 5. The two pneumonia classifications (bacterial and viral) appear to perform worse than the other classes, resulting in poor accuracy. The accuracy improves considerably when the two pneumonia courses are consolidated into one pneumonia class. This can be achieved with C19D-Net as presented in Table 6. The table represents that COVID detection continues to have the finest presentation when associated with the other two models. Finally, the result of the two-class classification with two models is shown in Table 7. The F1-score with the C19D-Net model is the highest among all the classes.

Since the publication of a dataset, researchers have been studying chest XR pictures for the correct expectation of COVID-19 infection. Following that, various attempts to construct a viable diagnosis model employing DL approaches were made. With CNN-based networks, the concept of TL has been widely applied. Most of the older methods, on the other hand, were tested with a restricted amount of data. 

**Figure 8:**
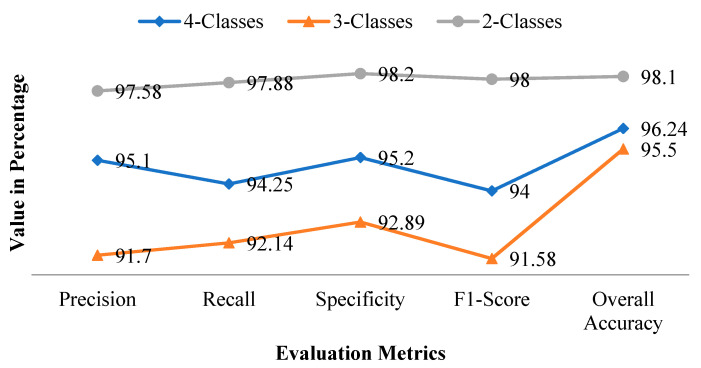
Metrics graph of all three classes.

The graphical performance of all four classes is shown in Figure 8 in terms of precision, F1-score, recall, and specificity. The precision calculates the total positive values in terms of total overall values (both true and positive). The precision of the above three classes (four-classes, three-classes, and two-classes) will change with the number of images. As with the precision the specificity increases which shows direct relation. The recall calculates the positive value in terms of both true positive and negative values. The F1-score calculates the two times of precision and recall value with total positive and total negative values. The model will be trained with a better number of images and shows the model specificity with an increase of images.

**Figure 9:**
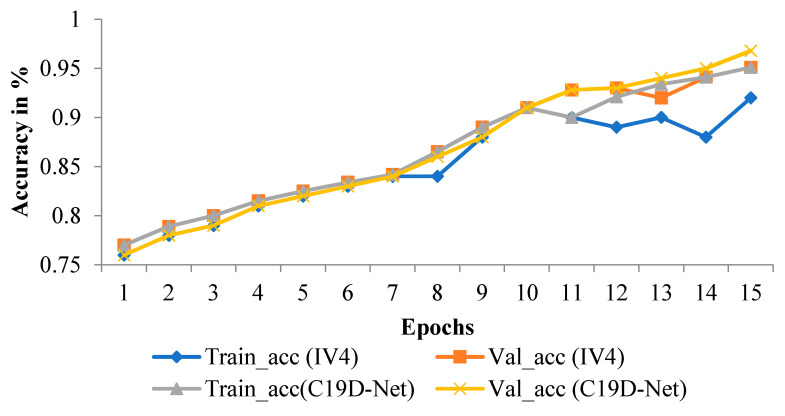
Accuracy graph of proposed C19D-Net with IncepttionV4.

The graphical representation of the proposed C19D-Net model accuracy is shown in Figure 9 in comparison with InceptionV4. The accuracy will increase and maintain constant after epoch 11. However, with InceptionV4 the accuracy increase or decrease after some point. As compared with pre-defined InceptionV4 models shown in the Figure the accuracy of C19D-net does not dropdown.

The yellow line of train_acc (C19D-Net model) will increase with the number of epochs. Before 10 epochs the accuracy increase in a small amount, but decrease at epoch 11 and after 11 the accuracy increases till the last epoch with maximum value. The blue line of train_acc (IV4 model) will increase or decrease before reaching a maximum value.

## 4. Discussion

The comparison and performance of twelve different models with the proposed “C19D-Net” model are indicated in Table 9. We can see that a variety of strategies for detecting COVID-19 have been offered by researchers from throughout the world. Some authors suggested different deep learning models such as CNN [32,41] with a different number of classes and number of images, with AlexNet and LeNet [41], nCOVNet [42], Inception Transfer Learning [43]. Some uses proposed model DeCoVNet [44], COVID-Net [45], ResNet + CNN [29], DenseNet121 [46] with a different number of layers and number of parameters for the diagnosis of disease using CT images, CXR images. Manual disease detection and categorization take more time, the pathologists may encounter challenges with observer variability while grading the various diseases. Various ways for classifying COVID-19 from XR photos have recently been presented by several researchers. To identify COVID-19 from patient chest XR, the majority of studies use image processing methods. Some of the existing frameworks are designed for four-class classification, while others are designed for two-class, three-class, and multi-class classification. The findings demonstrate the suggested superiority of the C19D-Net model in terms of multi-class classification, better accuracy achieved in classification. Furthermore, as shown in the table, the proposed C19D-Net outperformed in comparison with other existing deep learning studies that used chest XR images for the detection of COVID-19. In terms of classification accuracy, for binary, three-class, and four-class cases, the performance values of each research are presented in the table.

The CoroDet [15] model is based on a 22-layer in which nine layers are of Conv2d and nine are of maxpool2d layers. This model used the COVID-19 dataset which contains 500 COVID-19, 800 normal, 400 bacterial pneumonia, and 400 viral pneumonia images. While the CovXNet uses depth-wise convolution with different dilation rates to extract features efficiently, the stacking approach can be used to improve the accuracy of its predictions. The dataset’s CovXNet model had 305 CRIs from each class, which was utilized to diagnose COVID-19 infection. The C19D-Net model achieves better accuracy with 400 COVID-19 images, 600 normal, 450 bacterial pneumonia, and 450 viral pneumonia images.

## 5. Conclusions and Future Scope

COVID-19 has had a significant negative impact on our daily life, ranging from public health to the global economy due to forced lockdowns. In this paper, a deep learning C19D-Net model is proposed for exploiting chest XR images to detect and classify COVID-19 and other kinds of pneumonia infection. For classification matching, this system uses efficient deep features extractor InceptionV4 and MSVM. With four-classes, three-classes, and two-classes, the proposed system achieved an accuracy of 96.24%, 95.50%, and 98.1% respectively. The proposed C19D-Net system achieves the highest COVID detection performance when compared to InceptionV4 and other studies in the literature. As a result, the proposed method can be helpful as a model to overcome a lack of detecting resources in many nations afflicted by COVID-19 RT-PCR kits to assist the radiologists to maintain their detection accuracy. The performance of the proposed method outperforms existing state-of-the-art methods. Our C19D-Net model is supported by empirical evidence. The model’s results are presented and discussed well for its acceptance and better performance. Our long-term goal is to overcome hardware restrictions by training our proposed C19D-Net technique on larger image sets and comparing its performance to a wider number of existing methods. More images in the training stage are expected to assist us to improve the model’s performance in the future. Then lesser time-consuming methods would be needed in the future for the detection of COVID-19 patients for better treatment offering at an early stage.

## Figures and Tables

**Table 1:** Comparison of competitive recent related works.

Ref No.	Dataset Name	No. of Images Used	Pre-Processing Techniques	Architecture Mode	Performance Accuracy
[24]	Chest X-Ray	550	“Rescaling”	7 pre-trained CNNs VGG19, ResNetV2, DenseNet201, InceptionV3, InceptionResNetV2, Xception, and MobileNetV2	Accuracy = 90% Precision = 83% F1-Score = 91%
[25]	Chest X-Ray	740	“DA”, “Histogram”, “Feature Extraction” using “AlexNet”, K-means”, “PCA”	2pre-trained CNNs: ImageNet and ResNet	Accuracy = 95.12% Sensitivity = 97.91% Specificity = 91.87%
[26]	CT-Scans	1106	Segmentation	ResNet-101 ResNet-50 DenseNet-169 DenseNet-201	Accuracy = 94.9%
[27]	CT-Scans	381	NA	AlexNet, GoogleNet, DenseNet, Inception, ResNet, VGG, XceptionNet, and InceptionResNet	Accuracy = 95.33% Sensitivity = 95.33% F1-Score = 95.34%
[28]	Chest X-Ray	983	Data Augmentation	Convolutional Neural Network	Accuracy = 93.3% Sensitivity = 91%
[29]	Computed tomography images (CT)	618	Data Augmentation	ResNet-18	Accuracy = 86.7%
[30]	Chest X-Ray	940	Data Augmentation	Inception Architecture	Accuracy = 96% F1-score = 96% AUC = 95%
[31]	CXR	5856	NA	AlexNet	Accuracy = 93% Sensitivity = 89.18% Specificity = 98.92%
[32]	Computed tomography images (CT)	1000	NA	Convolutional Neural Network	Accuracy = 90%
Proposed C19-Net (Discussed in Section 4 in detail)	Chest X-Ray	1900	“Resizing”	InceptionV4 + Support Vector Machine	Accuracy = 96.24%

**Table 2:** Dataset summary of classification for four-classes.

Class	Images Count
COVID-19	400
Bacterial Pneumonia	450
Viral Pneumonia	450
Normal	600

**Table 3:** Dataset summary of classification for three-classes.

Class	Images Count
COVID-19	400
Pneumonia	900
Normal	600

**Table 4:** Dataset summary of classification for two-classes.

Class	Images Count
Normal	600
COVID-19	400

**Table 5:** Comparison based on the performance of four-class C19D-Net, InceptionV4.

Method	Classes	Precision	Recall	Specificity	F1-Score
C19D-Net	COVID-19	95.81	95.14	94.18	94.89
	Bacterial Pneumonia	95.89	94.28	94.88	94.72
	Viral Pneumonia	94.78	94.14	96.25	93.14
	Normal	95.71	95.84	95.88	95.12
Inception V4	COVID-19	94.85	94.36	95.02	95.18
	Bacterial Pneumonia	95.10	94.80	94.12	94.20
	Viral Pneumonia	95.82	95.25	94.28	95.88
	Normal	94.87	94.66	95.28	96.20

**Table 6:** Evaluation of three-class C19D-Net, InceptionV4.

Method	Classes	Precision	Recall	Specificity	F1-Score
C19D-Net	COVID-19	94.57	94.14	93.89	92.78
	Pneumonia	94.57	95.70	94.48	92.42
	Normal	95.25	95.44	92.47	91.25
Inception V4	COVID-19	95.25	90.14	93.47	94.00
	Pneumonia	94.48	91.25	93.42	95.00
	Normal	93.14	92.02	94.36	92.13

**Table 7:** Performance comparison of 2-class C19D-Net, InceptionV4.

Method	Classes	Precision	Recall	Specificity	F1-Score
C19D-Net	Non-COVID	96.51	97.1	97.45	98
	COVID-19	97.1	96.88	97.2	97.45
Inception V4	Non-COVID	96.1	97.14	96.80	97.8
	COVID-19	96.91	95.00	96.51	97.1

**Table 8:** Performance of different classes (four-class, three-class, two-class) using the C19D-Net Model.

Class Name	Precision	Recall	Specificity	F1-Score	Overall Accuracy
4-Classes	95.1	94.25	95.2	94.0	96.24
3-Classes	91.7	92.14	92.89	91.58	95.50
2-Classes	97.58	97.88	98.2	98	98.1

**Table 9:** Classification accuracy of proposed C19D-net model with other existing models.

Study (Ref)	Model	No. of Images	2-Class Accuracy	3-Class Accuracy	4-Class Accuracy
[32]	CNN + MODE	100,100	93.5	--	--
[41]	LeNet and AlexNet	25,25	95.38	--	--
[42]	nCOVNet	215,280	88.1	--	--
[43]	Inception Transfer Learning	195,258	82.9	--	--
[44]	DeCoVNet	313,229	90.8	--	--
[16]	CNN	224,700,504	--	93.48	--
[45]	COVID-Net	53,5526,8066	--	92.42	--
[29]	ResNet + CNN	219,224,175	--	86.72	--
[46]	DenseNet121	179,179,179	--	88.91	--
[6]	GoogleNet, AlexNet, DenseNet201	127,127,127	91.44	91.73	--
[15]	CoroDet	500,400,400,800	99.11	94.21	91.27
[47]	CovXNet	305,305,305,305	97.40	89.60	90.21
Proposed C19D-Net	InceptionV4 + SVM Classifier	400,450,600,450	98.1	95.50	96.24

## Data Availability

Not applicable.

## References

[1] Deeks J.J., Dinnes J., Takwoingi Y., Davenport C., Spijker R., Phillips S.T., Adriano A., Beese S., Dretzke J., Ruffano L.F. (2020). Anti-body tests for identification of current and past infection with SARS-CoV-2. Cochrane Database Syst. Rev..

[2] Dastider A.G., Sadik F., Fattah S.A. (2021). An integrated autoencoder-based hybrid CNN-LSTM model for COVID-19 severity prediction from lung ultrasound. Comput. Biol. Med..

[3] Serte S., Demirel H. (2021). Deep learning for diagnosis of COVID-19 using 3D CT scans. Comput. Biol. Med..

[4] Ozyurt F., Tuncer T., Subasi A. (2021). An automated COVID-19 detection based on fused dynamic exemplar pyramid feature extraction and hybrid feature selection using deep learning. Comput. Biol. Med..

[5] Gu J., Yang L., Li T., Liu Y., Zhang J., Ning K., Su D. (2021). Temporal relationship between serial RT-PCR results and serial chest CT imaging, and serial CT changes in coronavirus 2019 (COVID-19) pneumonia: A descriptive study of 155 cases in China. Eur. Radiol..

[6] Elkorany A.S., Alsharkawy Z.F. (2021). COVIDetection-Net: A tailored COVID-19 detection from chest radiography images using deep learning. Optik.

[7] Li J., Zhao G., Tao Y., Zhai P., Chen H., He H., Cai T. (2021). Multi-task contrastive learning for automatic CT and X-ray diagnosis of COVID-19. Pattern Recognit..

[8] Das A.K., Kalam S., Kumar C., Sinha D. (2021). TLCoV—An automated Covid-19 screening model using Transfer Learning from chest X-ray images. Chaos Solitons Fractals.

[9] Wu X., Chen C., Zhong M., Wang J., Shi J. (2021). COVID-AL: The diagnosis of COVID-19 with deep active learning. Med. Image Anal..

[10] Yang D., Xu Z., Li W., Myronenko A., Roth H.R., Harmon S., Xu S., Turkbey B., Turkbey E., Wang X. (2021). Federated semi-supervised learning for COVID region segmentation in chest CT using multi-national data from China, Italy, Japan. Med. Image Anal..

[11] Nour M., Cömert Z., Polat K. (2020). A Novel Medical Diagnosis model for COVID-19 infection detection based on Deep Features and Bayesian Optimization. Appl. Soft Comput..

[12] Demir F. (2021). DeepCoroNet: A deep LSTM approach for automated detection of COVID-19 cases from chest X-ray images. Appl. Soft Comput..

[13] Brunese L., Mercaldo F., Reginelli A., Santone A. (2020). Explainable Deep Learning for Pulmonary Disease and Coronavirus COVID-19 Detection from X-rays. Comput. Methods Programs Biomed..

[14] Das D., Santosh K.C., Pal U. (2020). Truncated inception net: COVID-19 outbreak screening using chest X-rays. Phys. Eng. Sci. Med..

[15] Hussain E., Hasan M., Rahman A., Lee I., Tamanna T., Parvez M.Z. (2020). CoroDet: A deep learning based classification for COVID-19 detection using chest X-ray images. Chaos Solitons Fractals.

[16] Apostolopoulos I.D., Mpesiana T.A. (2020). Covid-19: Automatic detection from X-ray images utilizing transfer learning with convolutional neural networks. Phys. Eng. Sci. Med..

[17] Kumar R., Arora R., Bansal V., Sahayasheela V.J., Buckchash H., Imran J., Narayanan N., Pandian G.N., Raman B. (2020). Accurate Prediction of COVID-19 using Chest X-Ray Images through Deep Feature Learning model with SMOTE and Ma-chine Learning Classifiers. MedRxiv.

[18] Wu X., Hui H., Niu M., Li L., Wang L., He B., Yang X., Li L., Li H., Tian J. (2020). Deep learning-based multi-view fusion model for screening 2019 novel coronavirus pneumonia: A multicentre study. Eur. J. Radiol..

[19] Quan H., Xu X., Zheng T., Li Z., Zhao M., Cui X. (2021). DenseCapsNet: Detection of COVID-19 from X-ray images using a capsule neural network. Comput. Biol. Med..

[20] Cruz J.F.H.S. (2021). An ensemble approach for multi-stage transfer learning models for COVID-19 detection from chest CT scans. Intell. Med..

[21] Xu Y., Lam H.K., Jia G. (2021). MANet: A two-stage deep learning method for classification of COVID-19 from Chest X-ray images. Neuro Comput..

[22] Verma P., Tripathi V., Pant B. (2021). Comparison of different optimizers implemented on the deep learning architectures for COVID-19 classification. Mater. Today Proc..

[23] Rahimzadeh M., Attar A. (2020). A modified deep convolutional neural network for detecting COVID-19 and pneumonia from chest X-ray images based on the concatenation of Xception and ResNet50V2. Inform. Med. Unlocked.

[24] Hemdan E.E.D., Shouman M.A., Karar M.E. (2020). COVIDX-Net: A Framework of Deep Learning Classifiers to Diagnose COVID-19 in X-Ray Images. arXiv.

[25] Abbas A., Abdelsamea M.M., Gaber M.M. (2020). DeTrac: Transfer Learning of Class Decomposed Medical Images in Convolutional Neural Networks. IEEE Access.

[26] Rohila V.S., Gupta N., Kaul A., Sharma D.K. (2021). Deep learning assisted COVID-19 detection using full CT-scans. Internet Things.

[27] Latif S., Usman M., Manzoor S., Iqbal W., Qadir J., Tyson G., Castro I., Razi A., Boulos M.N.K., Weller A. (2020). Leveraging Data Science to Combat COVID-19: A Comprehensive Review. IEEE Trans. Artif. Intell..

[28] Khalifa N.E.M., Taha M.H.N., Hassanien A.E., Elghamrawy S. (2020). Detection of Coronavirus (COVID-19) Associated Pneumonia based on Generative Adversarial Networks and a Fine-Tuned Deep Transfer Learning Model using Chest X-ray Dataset. arXiv.

[29] Xu X., Jiang X., Ma C., Du P., Li X., Lv S., Yu L., Ni Q., Chen Y., Su J. (2020). A Deep Learning System to Screen Novel Coronavirus Disease 2019 Pneumonia. Eng. J..

[30] Panahi A.H., Rafiei A., Rezaee A. (2021). FCOD: Fast COVID-19 Detector based on deep learning techniques. Inform. Med. Unlocked.

[31] Ibrahim A.U., Ozsoz M., Serte S., Al-Turjman F., Yakoi P.S. (2021). Pneumonia Classification Using Deep Learning from Chest X-ray Images during COVID-19. Cogn. Comput..

[32] Singh D., Kumar V., Kaur M. (2020). Classification of COVID-19 patients from chest CT images using multi-objective differential evolution–based convolutional neural networks. Eur. J. Clin. Microbiol. Infect. Dis..

[33] Fathollahi-Fard A.M., Hajiaghaei-Keshteli M., Tavakkoli-Moghaddam R., Smith N.R. (2021). Bi-level programming for home health care supply chain considering outsourcing. J. Ind. Inf. Integr..

[34] Fathollahi-Fard A.M., Woodward L., Akhrif O. (2021). Sustainable distributed permutation flow-shop scheduling model based on a triple bottom line concept. J. Ind. Inf. Integr..

[35] Fathollahi-Fard A.M., Hajiaghaei-Keshteli M., Mirjalili S. (2019). A set of efficient heuristics for a home healthcare problem. Neural Comput. Appl..

[36] Bahadori-Chinibelagh S., Fathollahi-Fard A.M., Hajiaghaei-Keshteli M. (2019). Two Constructive Algorithms to Address a Multi-Depot Home Healthcare Routing Problem. IETE J. Res..

[37] Shi Y., Zhou Y., Ye W., Zhao Q.Q. (2020). A relative robust optimization for a vehicle routing problem with time-window and synchronized visits considering greenhouse gas emissions. J. Clean. Prod..

[38] Dauletova M., Hafsan H., Mahhengam N., Zekiy A.O., Ahmadi M., Siahmansouri H. (2021). Mesenchymal stem cell alongside exosomes as a novel cell-based therapy for COVID-19: A review study. Clin. Immunol..

[39] (2020). Joseph Paul Cohen, Paul Morrison, Lan Dao, COVID-19 Image Data Collection. https://github.com/ieee8023/covid-chestxray-dataset.

[40] Kaggle P.M. (2020). Chest X-ray Images (pneumonia) Dataset. https://www.kaggle.com/paultimothymooney/chest-xray-pneumonia.

[41] Zhao X., Liu L., Qi S., Teng Y., Li J., Qian W. (2018). Agile convolutional neural network for pulmonary nodule classification using CT images. Int. J. Comput. Assist Radiol. Surg..

[42] Panwar H., Gupta P., Siddiqui M.K., Morales-Menendez R., Singh V. (2020). Application of deep learning for fast detection of COVID-19 in X-Rays using nCOVnet. Chaos Solitons Fractals.

[43] Wang S., Kang B., Ma J., Zeng X., Xiao M., Guo J., Cai M., Yang J., Li Y., Meng X. (2021). A deep learning algorithm using CT images to screen for Corona virus disease (COVID-19). Eur. Radiol..

[44] Zheng C., Deng X., Fu Q., Zhou Q., Feng J., Ma H., Liu W., Wang X. (2020). Deep Learning-based Detection for COVID-19 from Chest CT using Weak Label. MedRxiv.

[45] Wang L., Wong A. (2020). COVID-Net: A tailored deep convolutional neural network design for detection of COVID-19 cases from chest X-ray images. Sci. Rep..

[46] Li X., Zhu D. (2020). COVID-Xpert: An AI Powered Population Screening of COVID-19 Cases Using Chest Radiography Images. arXiv.

[47] Mahmud T., Rahman A., Fattah S.A. (2020). CovXNet: A multi-dilation convolutional neural network for automatic COVID-19 and other pneumonia detection from chest X-ray images with transferable multi-receptive feature optimization. Comput. Biol. Med..

